# Zingerone Suppresses Liver Inflammation Induced by Antibiotic Mediated Endotoxemia through Down Regulating Hepatic mRNA Expression of Inflammatory Markers in *Pseudomonas aeruginosa* Peritonitis Mouse Model

**DOI:** 10.1371/journal.pone.0106536

**Published:** 2014-09-03

**Authors:** Lokender Kumar, Sanjay Chhibber, Kusum Harjai

**Affiliations:** Department of Microbiology, BMS Block, Panjab University, Chandigarh, India; Fox Chase Cancer Center, United States of America

## Abstract

Antibiotic-induced endotoxin release is associated with high mortality rate even when appropriate antibiotics are used for the treatment of severe infections in intensive care units. Since liver is involved in systemic clearance and detoxification of endotoxin hence it becomes a primary target organ for endotoxin mediated inflammation. Currently available anti-inflammatory drugs give rise to serious side effects. Hence, there is an urgent need for safe and effective anti-inflammatory therapy. It is likely that anti-inflammatory phytochemicals and neutraceutical agents may have the potential to reduce the endotoxin mediated inflammation and complications associated with endotoxin release. Keeping this in mind, the present study was planned to evaluate the hepatoprotective potential of zingerone (active compound of *zingiber officinale*) against liver inflammation induced by antibiotic mediated endotoxemia. The selected antibiotics capable of releasing high content of endotoxin were employed for their *in vivo* efficacy in *P.aeruginosa* peritonitis model. Released endotoxin induced inflammation and zingerone as co-anti-inflammatory therapy significantly reduced inflammatory response. Improved liver histology and reduced inflammatory markers MDA, RNI, MPO, tissue damage markers (AST, ALT, ALP) and inflammatory cytokines (MIP-2, IL-6 and TNF-α) were indicative of therapeutic potential of zingerone. The mechanism of action of zingerone may be related to significant inhibition of the mRNA expression of inflammatory markers (TLR4, RelA, NF-kB2, TNF- α, iNOS, COX-2) indicating that zingerone interferes with cell signalling pathway and suppresses hyper expression of cell signaling molecules of inflammatory pathway. Zingerone therapy significantly protected liver from endotoxin induced inflammatory damage by down regulating biochemical as well as molecular markers of inflammation. In conclusion, this study provides evidence that zingerone is a potent anti-inflammatory phytomedicine against hepatic inflammation induced by antibiotic mediated endotoxemia. These results thus suggest that zingerone treatment can be used as a co-therapy with antibiotics to reduced endotoxin induced inflammation during treatment of severe *P.aeruginosa* infections.

## Introduction

Gram negative nosocomial pathogen *Pseudomonas aeruginosa* causes a variety of infections including spontaneous bacterial peritonitis pyogenic liver abscess, sepsis and septic shock [1], [2], [3]. Endotoxin, which is a hydrophobic glycolipid, is known to play a very imperative role in pathogenesis of *P. aeruginosa* mediated infections [4], [5], [6]. It is well recognized that cell free endotoxin is significantly more biologically functional than cell bound endotoxin and antibiotics, particularly those that act as inhibitors of cell wall biosynthesis, induce enormous amount of endotoxin release during treatment [7]. Plenty of experimental evidences from *in vitro, in vivo* and *ex vivo* models have advocated that antibiotics vary in their ability to trigger endotoxin release from gram-negative microbes [7], [8], [9]. Further, *ex vivo* evaluation of whole mouse blood has established that there is a correlation between amount of endotoxin release following antibiotic exposure and pro-inflammatory cytokine production [7].

Though liver is known to detoxify endotoxin but at the same time it also responds energetically to endotoxin leading to endotoxin induced inflammations. In liver, LBP (endotoxin binding protein) binds to endotoxin and activates CD14, toll-like receptor (TLR) 4 and MD2 surface receptor complex of macrophages, monocytes, hepatocytes and kupffer cells [10] resulting in potent inflammatory response. Endotoxin binds with TLR4 receptor which is highly expressed in cells that respond to endotoxin, such as macrophages, monocytes, hepatocytes and kupffer cells and induces expression of inflammatory genes through TLR4/NF-kB signaling pathway. NF-κB family consists of five structurally related proteins known as Rel/NF-κB proteins; p50, p52, RelA, RelB, and c-Rel [11]. Two signaling pathways are involved in the activation of NF-κB family. Canonical pathway (classical) and non-canonical pathway (Alternative) [12]. Canonical signaling pathway includes toll-like receptor super family which is helpful in recruitment of adaptor molecules such as TRAF (TNF Receptor Associated Factor) to cytoplasmic domain of the receptor. The canonical pathway induction involves RelA, RelB, c-Rel and p50 proteins to activate NF-κB [13]. In the non-canonical pathway, ligand induced activation of NF-κB is due to activation of NFkB-2, leading to liberation of p52/RelB [14]. Both these pathways activate transcription of array of different genes. TLR4 may have a role in non-canonical NF-κB signaling since its ligand (endotoxin) induces P100 processing in a B-cell line [15]. Further NF-κB regulates the production of pro-inflammatory mediators, such as TNF-α, COX-2 and iNOS and IL-12 which are mainly responsible for endotoxin induced tissue injury.

Till now antibiotic therapy is the most viable therapeutic choice which causes rapid killing of pathogen and quick recovery of infection. But it also leads to antibiotic induced endotoxin release which then interacts with humoral and cell mediated immune system to stimulate release of an array of inflammatory molecules leading to severe inflammation, fever, tissue injury and organ dysfunction [16], [17]. Hence, there is an urgent requirement for antibiotic-anti-inflammatory co therapy, selecting those antibiotics that will not only kill the pathogen instantly but also suppress the detrimental effects of endotoxin mediated inflammation. Current anti-inflammatory chemotherapy fails because of a number of side effects on cardiovascular, gastrointestinal and circulatory system. Therefore, therapy with no side effects might provide a hope for the suppression of inflammation induced by antibiotic mediated endotoxemia.

Herbal plant like *Zingiber officinale* is a natural dietary spice with potent anti-inflammatory, antioxidative and anticancer properties [18]. Zingerone [4-(4-hydroxy-3-methoxyphenyl) butan-2-one] is a stable active component of dry ginger rhizome [19] and has been found to down regulate age related activation of proinflammatory enzymes [20]; protect human lymphocytes from radiation induced genetic damage and apoptosis [21] reduce endotoxin induced acute lung injury in mice [22]. To the best of our knowledge not many studies are available on its *in vivo* protective effect against hepatic inflammation induced by antibiotic mediated endotoxemia. Keeping this in perspective, the aim of the present study was to assess the protective effect of zingerone on endotoxin induced liver damage in terms of liver histology, serum endotoxin levels and malondialdehyde (MDA), myeloperoxidase (MPO), nitrogen intermediates (RNI) and pro-inflammatory cytokine levels in liver homogenate. Effect of zingerone on endotoxin induced mRNA expression of inflammatory markers (TLR4, RelA, NF-κB2, TNF-α, iNOS and COX-2) was also evaluated in detail following *P.aeruginosa* induced peritonitis in mouse model of liver infection.

## Materials and Methods

### Ethical Statement

The experimental protocols were approved by the Institutional Animal Ethics Committee (Approval ID: IAEC/96) of Panjab University, Chandigarh, India and performed in accordance with the guidelines of Committee for the Purpose of Control and Supervision of Experiments on Animals (CPCSEA), Government of India. All efforts were made to minimize the suffering of animals.

### Bacterial strain

Standard strain *Pseudomonas aeruginosa* PAO1 was obtained from Dr. Barbara H. Iglewski, Department of Microbiology and Immunology, University of Rochester, New York, USA and maintained in nutrient agar stabs at 4°C.

### Drugs and chemicals

Pure zingerone [4-(4-hydroxy-3-methoxyphenyl) butan-2-one] was obtained from Gogia Chemical Industries, India. Antibiotics were purchased from Himedia chemicals, India. All other reagents and chemicals used were of analytical grade.

### Antibiotic susceptibility of PAO1

Antibiotic susceptibility of PAO1 against ciprofloxacin, amikacin, gentamicin and cefotaxime was tested by the standard broth dilution method according to the guidelines of the National Committee for Clinical Laboratory Standard.MIC values for all the antibiotics were calculated.

### Screening of antibiotics against PAO1 in terms of bacterial killing and endotoxin release *in vitro*

PAO1 was incubated at 37°C for 1.5, 3, 4.5 and 6 h in the presence of antibiotics (2 X MIC). Culture without antibiotics served as control to evaluate bacterial killing and endotoxin release.

### Bacterial killing and endotoxin release

To qantitate bacterial number, samples were taken at different time intervals and serially diluted in phosphate buffer saline and spread plated to MacConkey agar plates. Colonies were counted after overnight incubation at 37°C. The amount of cell free endotoxin in these samples was determined after removing bacteria by passing cell free supernatant through 0.22 µm Millipore filters. 0.1 ml sample was incubated with 0.1 ml Limulus amebocyte lysate (LAL) (GenScript USA) at 37°C. Absorbance was measured at 545 nm spectrophotometrically. The endotoxin levels were calculated against a standard curve of pure endotoxin of *E. coli* as per manufacturer's instructions.

### Protective effect of zingerone on antibiotic mediated endotoxemia against *Pseudomonas aeruginosa* peritonitis in a murine model

BALB/c mice of either sex (8–10 week-old; 20–30 g) were procured from Central Animal House Panjab University Chandigarh. Animals were allowed free access to food and water at all times and were maintained in a controlled temperature (20–25°C) and humid (50±5%) environment. A total of 6 groups having 16 mice in each group were used in duplicate. Mice were infected intraperitoneally with 500 µl of *P.aeruginosa* cells (10^5^ cfu/ml) to establish *P.aeruginosa* induced peritonitis, experimental model of liver infection. On the peak day of infection (5^th^ day) mice were administered with single dose of cefotaxime and amikacin intramuscularly. Cefotaxime at a concentration of 100 mg/kg body weight and amikacin at 75 mg/kg body weight of mice administered to achieve high serum concentration necessary for a rapid bactericidal action. In antibiotic-zingerone groups, mice were administered single dose of zingerone (100 mg/kg body weight) immediately after antibiotic administration. Zingerone dose selected was 100 mg/kg approximately corresponds to 1/10th of LD_50_ [23]. PAO1 infected mice receiving normal saline served as control. After 0, 1.5, 3, 4.5, 6 h of antibiotic exposure, mice were sacrificed, blood was collected by retro-orbital puncture in two aliquots and serum was separated and liver was removed aseptically. Liver tissue homogenate and serum samples were stored at −60°C till analysis was carried out.

### Histopathological examination

Liver tissue samples fixed in 10% buffered normal saline and dehydrated in 30–100% gradient ethanol. Paraffin wax blocks were prepared and 5 µ thin sections were stained with hematoxylin eosin and Masson's trichrome stain. Liver sections were examined for inflammatory response and liver fibrosis.

### Serum endotoxin levels

LAL Endotoxin Assay Kit (GenScript USA Inc.) was used for detection of endotoxin levels in serum samples. Briefly, 0.1 ml serum was incubated with 0.1 ml Limulus amebocyte lysate (LAL) at 37°C. Absorbance was measured at 545 nm spectrophotometrically.

### Preparation of tissue homogenate

Liver tissue was harvested, washed in ice cold physiological saline and homogenized in buffer using glass homogenizer to obtain 10% homogenate. The tissue homogenate was centrifuged at 12,000 X g for 10 minutes at 4°C and the supernatant was collected.

### Bacteriological examination

To qantitate bacterial numbers, liver homogenate samples taken at different time intervals were serially diluted in phosphate buffer saline (PBS pH 7.2) and 0.1 ml from each dilution was spread plated on to MacConkey's agar plates. Colonies were counted after overnight incubation at 37°C.

### Biochemical analysis of liver homogenates for the production of inflammatory mediators

#### Malondialdehyde (MDA) estimation

Induction of pathology was evaluated on the basis of Malondialdehyde, the index of lipid per oxidation following the method of Anjaneyulu and Chopra.,[24]. Briefly, tissue homogenate was added to tris HCl followed by the addition of ice-cold trichloroacetic acid. Supernatant was taken and mixed with thiobarbituric acid. Tubes were covered and kept in a boiling water bath for 10 min. After cooling, absorbance was read at 532 nm. The level of lipid peroxide was expressed as nmoles of MDA formed/mg of protein.

### Reactive nitrogen intermediates (RNI) estimation

Nitrite was estimated in the liver tissue of mice following the method of Rockett et al., [25]. Briefly, samples were mixed with Griess reagent (Sigma Aldrich Chemicals Ltd., St Louis, MO, USA) followed by addition of trichloroacetic acid and incubated at room temperature. After centrifugation, the optical density of supernatant was read at 540 nm.

### Myeloperoxidase (MPO) estimation

MPO activity was quantified by using the myeloperoxidase assay as described by Hang el al., [26]. Briefly, tissue was homogenized in potassium phosphate with hexadecyl trimethyl ammonium bromide and EDTA. The homogenate was sonicated and centrifuged. Supernatant was mixed with o-dianisidine and absorbance was read at 490 nm at 0 min, 1 min 2 min at room temperature to determine change in absorbance per minute. It was calculated by using the formula: MPO activity (U/mg)  =  X/weight of the piece of tissue taken, where X = 10 X change in absorbance per min/volume of supernatant taken in the final concentration.

### Estimation of TNF-α, MIP-2 and IL-6 cytokines by ELISA

Levels of pro-inflammatory cytokines (TNF-α, MIP-2 and IL-6) in liver homogenate were assessed by using ELISA kits (Peprotech USA) according to the manufacturer's instructions. Ninety-six-well microtiter plates (Falcon Corp., USA) were coated with 100 µl of a suitable capture antibody per well. The plates were coated with 100 µl of sample and incubated at room temperature for two hours. The plates were washed with wash buffer and incubated with streptavidin antibodies followed by incubation with biotinylated antibodies. Plates were incubated in the dark with TMB substrate after washing. Once adequate color developed ELISA plates were read at 405 nm using Microplate Manager 5.1 (BioRad Labs Ltd. USA). Cytokine levels were estimated by using the standard recombinant cytokine supplied along with the kits as a reference.

### Serum AST, ALT and ALP estimation

Aspartate aminotransferase (AST), Alanine aminotransferase (ALT), and alkaline phosphatase (ALP) enzyme activities in serum were determined using ERBA test kits (ERBA Diagnostics, Mannheim, Germany) at 6 h interval in different groups.

### Therapeutic potential of zingerone on endotoxin induced hepatic inflammation in terms of mRNA expression of inflammatory markers (TLR4/RelA/NF-kB2/TNF- α/iNOS/COX-2) *in vivo*

Group having 24 mice each (BALB/c 3–5 weeks old and weighing 20–30 gm) was put in duplicate. Purified endotoxin of *P.aeruginosa* PAO1 was administrated intraperitoneally (1 mg/kg body weight) and 6 mice each were sacrificed at 4, 8, 12 and 24 h. mRNA expression of the genes was evaluated in liver tissue using reverse transcription–polymerase chain reaction. To evaluate the therapeutic potential of zingerone in terms of production of mRNA of inflammatory genes, three groups of 6 mice each (BALB/c 3–5 weeks old and weighing 20–30 gm) in duplicate were used and were sacrificed at 8 h, as maximum mRNA expression was found at 8 h after LPS administration. In 1^st^ group endotoxin was administrated intraperitoneally (1 mg/kg body weight) and in 2^nd^ group the mice were administered one dose of zingerone (100 mg/ml) immediately after endotoxin treatment. Mice receiving normal saline served as controls. Level of mRNA expression of the genes was evaluated using reverse transcription–polymerase chain reaction.

### Reverse transcription–polymerase chain reaction (RT–PCR)

Nucleotide sequence for genes was taken from NCBI data base. For each gene primers were designed using Primer 3 online tool. Primer sequences used for PCR amplification of c DNA are mentioned in Table. 1. Liver tissue was homogenized with Trizol Reagent (Invitrogen USA). The homogenate was centrifuged at 3000 X g at 48°C for 10 min. The supernatant was mixed with chloroform and precipitated with 75% ethanol. The total amount of RNA was determined using the spectrophotometric analyzer, Nano Drop 100 (Thermo scientific). RNA was reverse-transcribed into cDNA using a First-Strand cDNA Synthesis kit (Fermetas USA) with oligo (dT) primer. The cDNA was amplified with specific primers for TLR-4, RelA, NFκB2, TNF-α, iNOS, COX-2 and GAPDH as a control. Sample was incubated using a MJ Minin PCR Thermal Cycler (Biorad USA) and ran 34 times. The cycles lasted for 30 S at 94°C, for 60 S at 58°C, and for 60 s at 68°C for RelA, NF-κB1 and cycles lasted for 30 S at 94°C, for 60 S at 56°C, and for 60 s at 68°C for Cox-2, iNOS,TLR4,TNF-α. For control gene GAPDH the cycles lasted for 30 S at 94°C, for 60 S at 55°C. The final incubation was at 72°C for 5 min. Amplified PCR products were separated electrophoretically on a 1.0% agarose gel, and bands were visualized with ethidium bromide under ultraviolet transillumination. Densitometry of PCR product to determine relative mRNA expression was performed by Gel Doc Multi-Analyst (BioRad USA).

**Table 1:** List of primer sequence for genes.

S.NO.	GENES	LEFT PRIMER	RIGHT PRIMER	PCR Product Size (bp)
1.	RelA	5′-GGCCTCATCCACATGAACTT-3′	5′-CACTGTCACCTGGAAGCAGA-3′	201
2.	NF-κB2	5′-ACCTTTGCTGGAAACACACC-3′	5′-ATGGCCTCGGAAGTTTCTTT-3′	245
3.	TLR4	5′-GCTTTCACCTCTGCCTTCAC-3′	5′-TGCCGTTTCTTGTTCTTCCT-3′	395
4.	TNF-α	5′-TATGGCTCAGGGTCCAACTC-3′	5′-AAGCAAAAGAGGAGGCAACA-3′	495
5.	iNOS	5′-AGACCTCAACAGAGCCCTCA-3′	5′-GAACCTCCAGGCACACAGTT-3′	263
6.	Cox-2	5′-CCCCCACAGTCAAAGACACT-3′	5′-AGGCAATGCGGTTCTGATAC-3′	348
7.	GAPDH	5′-AACTTTGGCATTGTGGAAGG-3′	5′-GGATGCAGGGATGATGTTCT-3′	132

### Statistical analysis

All experiments were performed in duplicate and repeated on different days. The effect of zingerone treatment on antibiotic induced endotoxemia and relative mRNA expression of genes in different treated groups with control was evaluated using two-way ANOVA test. p values were calculated and p<0.05 was considered significant. Data was analyzed using Graph Prism 5.0 software. Values were expressed as mean + S.E.M.

## Results

### Antibiotic susceptibility of PAO1

MIC values for ciprofloxacin, amikacin, gentamicin and cefotaxime against PAO1 were determined and found to be 0.3, 3.0, 30.0 and 25.0 µg/ml respectively.

### Effect of antibiotics on PAO1 in terms of bacterial killing and endotoxin release *in vitro*

**Figure 1:**
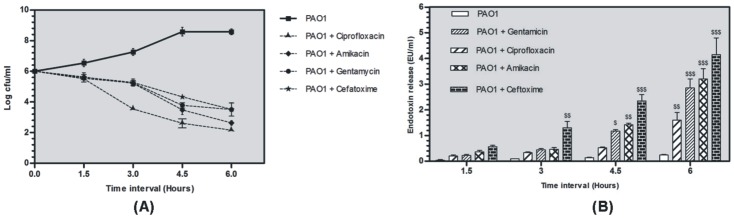
*In vitro* bacterial killing (Fig.1-A) and endotoxin release (Fig.1-B) potential of antibiotics against *P.aeruginosa* PAO1 ($ p<0.01, $ $ p<0.01 and $ $ $ p<0.001).

All antibiotics (2X MIC) showed decrease in viable counts and significant reduction was found at 6 h hour (p<0.001). Ciprofloxacin showed highest bactericidal action as compared to rest of the antibiotics (Fig.1 –A). Varied amount of cell free endotoxin was released on exposure to different antibiotics. Cefotaxime and amikacin were found to be efficient endotoxin releasing antibiotics and both the antibiotics significantly released high amount of endotoxin (p<0.001) (Fig.1 –B). On the basis of results from *in vitro* endotoxin release assay, cefotaxime and amikacin were selected for *in vivo* endotoxin release studies. Effect of zingerone was also evaluated for endotoxin release potential of antibiotics *invitro*. No significant effect was found (supplementary data) on the endotoxin levels indicating that zingerone did not interfere with the endotoxin release potential of antibiotics.

### Protective effect of zingerone on hepatic inflammation induced by antibiotic mediated endotoxemia in PAO1 infected BALB/c mice

#### Liver histology

**Figure 2:**
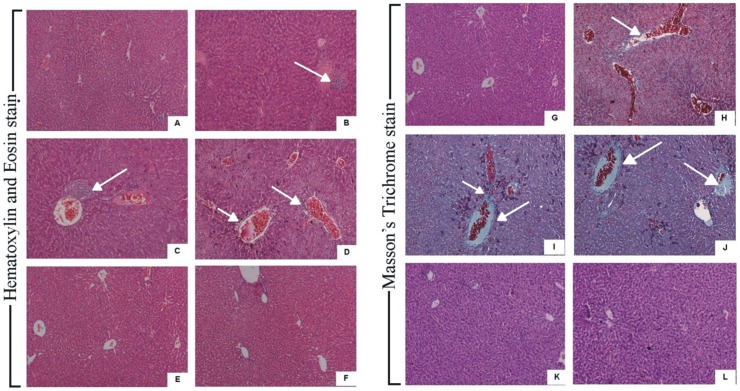
Liver tissue in antibiotic alone group showed high liver inflammatory response with infiltration of neutrophilic granulocytes (white arrow) indistinct boundaries between cytoplasm and nucleus of liver cells, hepatic portal haemorrhage and hepatocyte necrosis (white arrow) [Fig.2 (amikacin) C, I (cefotaxime) D, J] as compared to infection control (Fig.2 B, H). Uninfected group (control) did not show any sigh of inflammatory response (Fig.2 A, G). Amikacin-zingerone treatment (Fig.2 E, K) as well as cefotaxime-zingerone treatment (Fig.2 F, L) significantly protected mice from hepatic inflammation induced by antibiotic mediated endotoxemia and liver tissue appeared to be normal as was observed in control group (uninfected group).

Histological analysis of liver tissue obtained from antibiotic treated infected groups showed increased infiltration of neutrophilic granulocytes, necrosis of hepatocyte and hepatic portal inflammation along with hepatic portal haemorrhage and liver tissue fibrosis (Fig.2-C,I and Fig2-D,J) as compared to infection (PAO1) control (Fig.2-B,H). Mice without any infection did not show any inflammatory response (Fig.2-A, G). Cefotaxime-zingerone (Fig.2-E, K) as well as amikacin-zingerone (Fig.2-F, L) treatment showed very less neutrophil infiltration, no necrosis and portal haemorrhage in the liver tissue. The findings were comparable to normal as observed in control group.

#### Bacteriological examination

**Figure 3:**
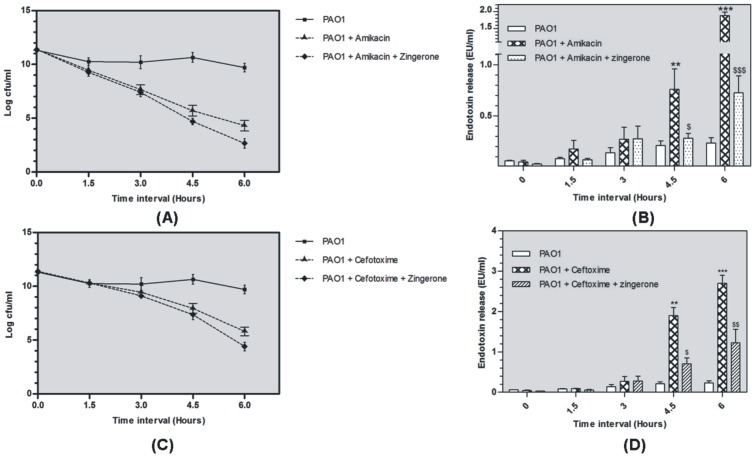
*In vivo* bacterial killing and endotoxin release potential of antibiotics against *P.aeruginosa* PAO1 [bacterial killing curve Fig.3 (amikacin-A, cefotaxime-C) and endotoxin release (Fig.3- amikacin-B, cefotaxime-D)] ($, * p<0.01, $ $, ** p<0.01 and ***, $ $ $ p<0.001) (*indicates comparison between infection control and antibiotic alone groups and $ indicates comparison between antibiotic alone and antibiotic-zingerone treated groups).

Mean decrease in bacterial count was achieved in the liver of mice following infection with *P.aeruginosa* along with antibiotic treatment at different time intervals (Fig.3). After amikacin therapy, a steady decrease in bacterial count was observed from 7.6 log cfu (3 h) to 4.3 log cfu (6 h) (Fig. 3 -A). Similar trend was observed with cefotaxime and the viable counts were 9.4 log cfu (3 h) and 5.8 log cfu (6 h) (Fig. 3 -C). Simultaneous administration of zingerone along with amikacin and cefotaxime did not show any further decrease in viable count of bacteria at all time intervals except at 6 h when significant difference was observed (p<0.05).

#### Serum Endotoxin Levels

Significantly high serum endotoxin levels were observed in PAO1 + Antibiotic group. With cefotaxime and amikacin, significant endotoxin release occurred between 3 to 4.5 h of exposure, reaching a maximum of 2.7 EU/ml and 1.88 EU/ml (p<0.001) for (Fig.3-B) cefotaxime and amikacin (p<0.001) respectively (Fig.3-D). Zingerone treatment significantly reduced the endotoxin levels at 3, 4.5 and 6 h. In cefotaxime and amikacin treated groups endotoxin levels were significantly reduced to 1.22 EU/ml and 0.72 EU/ml (p<0.01) respectively at 6 h.

### Production of inflammatory mediators

#### Malondialdehyde (MDA) estimation

**Figure 4:**
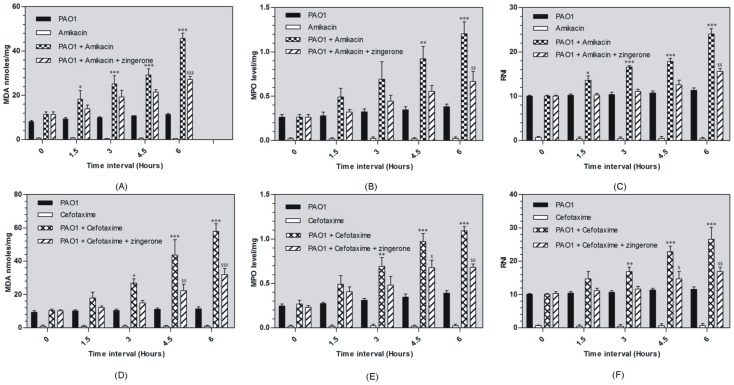
Effect of zingerone treatment on hepatic MDA/RNI/MPO production in liver homogenate against antibiotic mediated endotoxemia (amikacin Fig.4-A, B, C) and cefotaxime (Fig 4-D, E, E) ($, * p<0.01, $ $, ** p<0.01 and ***, $ $ $ p<0.001).

Liver homogenate of infected animals showed moderate amount of MDA but treatment with amikacin significantly increased MDA content and maximum increase was found at 6 h (45.66±3.4 nmoles/mg) (p<0.001) (Fig.4 A). Simultaneous treatment of amikacin with zingerone resulted in decrease in MDA content and significant decrease was found at 6 h (27.1±2.1 nmoles/mg) (p<0.001) (Fig.4 A). Similarly, cefotaxime increased MDA content significantly at all time intervals (p<0.001) (Fig.4 D). Simultaneous treatment of cefotaxime with zingerone decreased MDA content significantly at 4.5 h (p<0.01) and at 6 h (p<0.001) (Fig.4 D).

#### Myeloperoxidase (MPO) estimation

Treatment with amikacin increased MPO content initially but significant increase was found at 4.5 h and 6 h (p<0.001) (Fig.4 B). Zingerone treatment slightly decreased MPO at 3 and 4.5 h but significant decrease was found at 6 h (0.66±0.16 U/mg nmoles/mg) (p<0.01) (Fig.4 B). Similarly, cefotaxime significantly increased MPO content at all time intervals (p<0.001) (Fig.4 E). Zingerone treatment reduced MPO content and significant decrease was observed at 4.5 h and 6.0 h (p<0.01) (Fig.4 E).

#### Reactive nitrogen intermediates (RNI) estimation

Infected mice showed moderate amount of RNI but treatment with amikacin significantly increased RNI content with maximum increase seen at 6 h (p<0.001) (Fig.4 C). Following treatment with zingerone, slight decrease in RNI content was found at 3 and 4.5 h but significant decrease was found at 6 h (p<0.01) (Fig.4 C). Likewise, cefotaxime significantly increased RNI content at 3 h, 4.5 h and maximum increase was found at 6 h (26.59±5.11 nmoles/mg) (p<0.001) (Fig.4 F). With zingerone treatment RNI content decreased at 1.5, 3.0 and 4.5 h interval but significant reduction was found at 6 h (16.9±1.8 nmoles/mg) (p<0.01) (Fig.4 F).

#### Estimation of TNF-α, MIP-2 and IL-6 cytokines by ELISA

**Figure 5:**
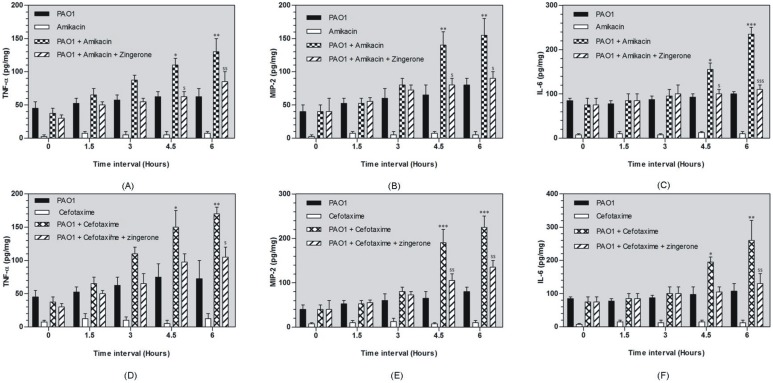
Effect of zingerone treatment on hepatic pro-inflammatory cytokine production (TNF-α, MIP-2 and IL-6) in liver homogenate against antibiotic mediated endotoxemia (cefotaxime Fig.5-A, B, C) and amikacin (Fig 5-D, E, E) ($, * p<0.01, $ $, ** p<0.01 and ***, $ $ $ p<0.001).

Amikacin and cefotaxime treatment led to decrease in bacterial load but significant increase in TNF-α, MIP-2 and IL-6 proinflammatory cytokines production was observed (Fig.5). After amikacin therapy levels of TNF-α, MIP-2 and IL-6 were significantly increased at 3 h, 4.5 h and with maximum increase observed at 6 h (Fig.5-D). Cefotaxime was found to be more effective in inducing production of proinflammatory cytokines. Significant increase of all the three cytokines was observed at 3 h, 4.5 h and 6 h (p<0.001) (Fig 5-A). Zingerone treated group showed decrease in the levels of proinflammatory cytokine at 1.5, 3, 4 h but significant difference was found only at 6 h. In amikacin + zingerone group, TNF-α levels were significantly decreased at 6 h (85 pg/mg) (p<0.01) (Fig 5-D). Zingerone treatment also decreased MIP-2 and IL-6 cytokine levels at 6 h (90 pg/mg) (p<0.05) and (110 pg/mg) (p<0.001) respectively (Fig 5-E, F). Zingerone was also able to suppress cytokines production after cefotaxime exposure at 6 h. The levels of TNF- α, MIP-2 and IL-6 were found to be 105 pg/mg (p<0.05), 135 pg/mg (p<0.01) and 130 pg/mg (p<0.01) respectively (Fig 5-A,B,C).

#### Serum AST, ALT and ALP levels

Control group without infection showed normal AST, ALT and ALP levels in serum (Table 2). Infection group showed elevated levels of these markers. Antibiotic treated groups showed comparatively high level of the tissue damage markers (Table 2). Cefotaxime treatment showed highest level of these enzymes. Interestingly zingerone as co-therapy significantly reduced AST, ALT and ALP levels indicating protective effect of zingerone against antibiotic induced liver damage (Table 2).

**Table 2:** Protective effect of zingerone on enzyme activities in serum (ALT, AST and ALP) against antibiotic induced endotoxemia after 6 hours on peak day of infection by *P.aeruginosa* PAO1.

Groups	ALT (IU/L)	AST (IU/L)	ALP (IU/L)
Control	16.16±3.69	27.99±3.30	87.87±10.40
PAO1	42.94±3.83	57.92±3.22	160.44±6.91
PAO1 + Amikacin	45.41±6.93	57.86±10.80	162.95±10.89
PAO1 + Cefotaxime	50.41±7.33	63.42±4.10	168.15±10.59
PAO1 + Amikacin + Zingerone	21.39±1.18	31.78±2.19	95.16±7.29
PAO1 + Cefotaxime + Zingerone	22.89±3.62	33.36±3.01	103.49±7.30

### Endotoxin induced liver inflammation in terms of mRNA expression of TLR4, RelA, NF-kB2, TNF- α, iNOS, COX-2 genes *in vivo*

#### Time dependent expression studies of gene expression in liver tissue against purified endotoxin

**Figure 6:**
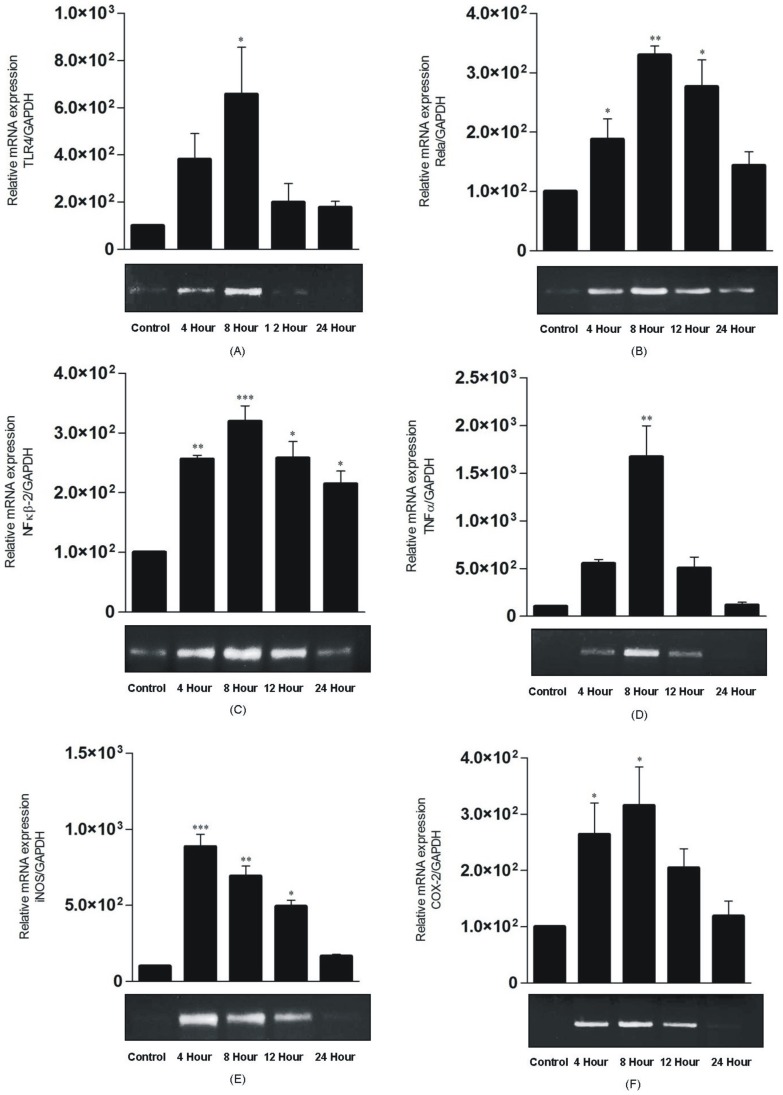
Effect of purified endotoxin on relative mRNA expression of TLR4, RelA, NF-kB2, TNF- α, iNOS, COX-2 genes (GAPDH as control gene) in liver tissue of mice (* P<0.05, ** p<0.01 and ** p<0.001).

**Figure 7:**
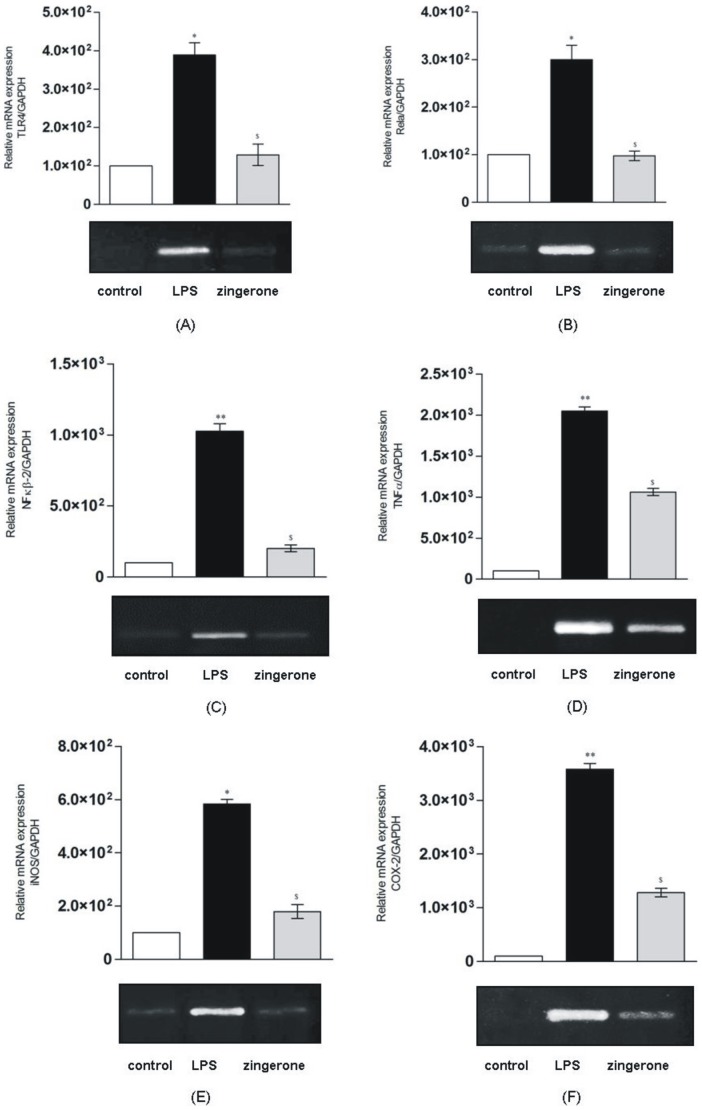
Effect of zingerone on the mRNA expression of inflammatory genes against endotoxin induced liver inflammation ($, * p<0.01, $ $, ** p<0.01 and ***, $ $ $ p<0.001).

Endotoxin administration caused potential increase in TLR4/NF-κB dependent expression of genes. TLR4 mRNA expression increase was time dependent. It started increasing at 4 h and was found to be maximum at 8 h (>7 folds) after which its expression declined (Fig. 6-A). Relative RelA mRNA expression was slightly increased at 4 h and maximum at 8 h (>3 folds) (Fig.6-B). Similarly, both NF-κB2 and COX-2 genes were expressed highest at 8 h (>3 folds) and declined later (Fig.6-C, F). Relative mRNA expression of proinflammatory cytokine TNF-α increased significantly at 4 h and reached its maximum level at 8 h (>15 folds) (Fig.6-D). iNOS gene expression was highest at 4 h (>8 folds) and remained active up to 8 h (>5 folds) decreasing thereafter leading to minimum level at 24 h (Fig. 6 B) (Fig.7-E). Results indicated maximum expression of most of the genes at 8 h interval in endotoxin treated group (Fig. 6 A and B). At 12 h, expression level of all the genes started to decline and at 24 h, minimum expression was observed (Fig6).

#### Effect of zingerone treatment on gene expression

Maximum expression of inflammatory markers was observed at 8 h after endotoxin administration, therefore protective effect of zingerone in term of gene expression was evaluated at 8 h only (Fig.7). Results showed that in endotoxin induced animals, zingerone treatment could reduce the mRNA expression of TLR4 by >2 fold (Fig.7-A). Similarly, mRNA expression of RelA and NF- κB2 was also found to be inhibited significantly (>1.5 folds and >5 folds respectively) (Fig.7-B, C). Relative mRNA expression level for TNF- α in zingerone treated group was significantly reduced (>2 folds) as compared to endotoxin treated animals (Fig.7-D). Specific inflammatory enzymes iNOS and COX-2 were found to be inhibited significantly (>3 folds and >5 folds respectively) (Fig.7-E, F) in zingerone treated animals. Results showed that post endotoxin treatment with zingerone significantly reduced (p≤0.05) mRNA expression of all these inflammatory markers in mice.

## Discussion

Correlation between endotoxin release and corresponding type/dose of antibiotic is well known and many *in vitro* and *in vivo* studies are available on this aspect [7], [9]. Antibiotics rapidly kill the pathogen and release enormous amount of endotoxin in blood stream. Different classes of antibiotics targeting cell wall, protein synthesis, pathway of DNA metabolism differ in their potential to release cell free endotoxin. In the present study, endotoxin releasing potential of ciprofloxacin, amikacin, gentamicin and cefotaxime was studied in *P.aeruginosa* PAO1. Endotoxin release with ciprofloxacin was least and maximum with cefotaxime on treating *P.aeruginosa* cells *in vitro.* Ciprofloxacin acts on the A subunit of DNA gyrase, which inhibits DNA supercoiling, resulting in the inhibition of DNA replication [27] without causing cell lysis. Amikacin and gentamicin that inhibit protein synthesis are also known to release low amounts of endotoxin as compared to beta lactam antibiotics [28]. Whereas, cefotaxime (7-[2-(2-amino-4-thiazolyl)-2-methoximino]-acetamido cephalosporanate) has high affinity for penicillin-binding proteins (PBPs) and induces formation of filamentous cells leading to cell lysis [29]. High endotoxin release in gram negative bacteria (*E.coli)* has also been linked to significantly high endotoxin level in plasma and IL-6 pro-inflammatory cytokines in serum [30]. Since, cefotaxime and amikacin were found to release high amounts of endotoxin as compared to gentamicin and ciprofloxacin hence these two antibiotics were selected for *in vivo* studies.

Immunostimulatory mechanism of *P. aeruginosa* in liver inflammation induced by antibiotic mediated endotoxemia is still not very well understood. Liver is responsible for detoxification of endotoxin from blood stream and is most susceptible to endotoxin mediated inflammatory damage [31]. During infection and even during antibiotic treatment, liver becomes the primary target organ for endotoxin stimulation. Endotoxin-TLR4 mediated signalling pathway enhances production of inflammatory mediators following *P.aeruginosa* infection [32]. Endotoxin-induced liver injury has been used as an experimental model to analyze the mechanism of endotoxin-induced liver inflammation using *E.coli* endotoxin [33], [34]. In the present study both cefotaxime and amikacin induced significant endotoxin release *in vivo.* To study this phenomenon *P. aeruginosa* induced peritonitis mouse model of liver infection was established. Animal group on peak day of infection were treated with high dose of either cefotaxime or amikacin. Liver inflammatory response was significantly high after 6 h of antibiotic administration and this was linked to high endotoxin release by antibiotics. This indicated that the high inflammatory response was induced by endotoxin release due to immediate lysis of bacteria and remained till the endotoxin was cleared from the organs and circulatory system completely. After 6 h inflammation was significantly reduced and infection treated completely in antibiotic treated group (data not shown). Biochemical analysis of liver homogenate for inflammatory mediators indicated elevated levels of MDA, MPO and RNI. Lipid peroxidation is well known marker for tissue destruction which indicates oxidative degradation of lipids and also indicative of inflammatory injury and tissue damage. Elevated MDA levels observed in this study indicated that the product of immediate lysis of bacteria caused stimulation of liver cells and generation of free radical damage that led to oxidative damage to cell membranes. Histopathological changes observed in tissue sections relate to reactive nitrogen intermediates (RNI) production, a potential source of free radical mediated inflammation or tissue damage. Since neutrophils are major effector cells in damaging the liver and an important source of free radicals [35], hence, enhanced MPO activity observed may have contributed to hepatocyte necrosis, proinflammatory cytokine production and hepatic inflammation. High myeloperoxidase activity is a marker of local and systemic inflammation [36], relating tissue destruction inflammatory response to bacterial antigens. Overzealous production of pro-inflammatory cytokines including TNF-α MIP-2 and IL-6 can result in shock, multi organ dysfunction, and even death [37]. In the past, over expression of MIP-2 protein has been specifically linked with endotoxin mediated hepatic injury [38]. Proinflammatory cytokines play a crucial role in endotoxin-induced liver injury leading to hepatotoxicity [39].TNF- α and IL-6 cytokine were found to be highly expressed in liver during inflammation as a result of endotoxemia [40].

Following zingerone treatment proinflammatory cytokines also showed significantly low levels (p<0.05). Anti-inflammatory activity of zingerone in this study, could be attributed to phenolic nature of zingerone which might have led to scavenging of free radicals [20]. Methoxy group with phenolic hydroxyl group in zingerone facilitates proton release along with long chain ethyl methyl ketone group providing bulk stabilization to zingerone molecule [21]. This may lead to cell penetration and scavenging of free radicals. Anti-inflammatory potential of zingerone treatment along with antibiotic therapy showed decrease in inflammatory response in terms of decreased neutrophilic granulocyte infiltration and no hepatic portal haemorrhage. Hepatic haemorrhage was also absent in zingerone treated liver tissue. Levels of Inflammatory mediators MDA, RNI and MPO in zingerone treated animals were also significantly reduced (p<0.05).

A significant body of evidence indicates that Injury by LPS particularly in liver involves LPS binding proteins (LBP) which activate the CD14/TLR4 receptor and in turn induce transduction of inflammatory signals resulting in the regulation of inflammatory mediator production[41]. Inflammatory markers chosen for the study have been found to play significant role in LPS *in vivo* induced tissue injury through NF-κB. Time dependent expression of genes induced by LPS revealed that expression of some genes started early at a time interval of 4 h (iNOS, NF-κB2) and some at 8 h (TLR4,TNF-α, RelA, and COX-2). Level of expression was found to be variable but maximum expression was found at 8 h. In the present study, *P.aeruginosa* LPS significantly enhanced mRNA expression of TLR4 receptor leading to increase in the number of TLR4 receptors on the liver cell surface. Due to this, more binding of LPS to cells resulting in potent induction of inflammatory response was observed. Zingerone treatment significantly reduced the level of mRNA expression of TLR4 receptor indicating reduced number of TLR4 receptors and thereby less binding of LPS. This may have led to decreased inflammatory response after zingerone treatment. During gram-negative sepsis, LPS induced cells are triggered to produce large quantities of pro-inflammatory cytokines such as tumor necrosis factor alpha (TNF-a) in response to endotoxin [42]. TNF-α is secreted by a variety of cells, including hepatocytes, kupffer cells mast cells and epidermal cells. However, mainly activating macrophages and natural killer cells, release potent biologically active substances which cause shock, fever, organ failure and other pathophysiological implications [43] Workers have also found that TNF-α plays a crucial role in LPS-induced liver injury leading to hepatotoxicity [39]. In the present study, LPS caused tremendous increase in TNF- α levels at 4 h and 8 h after LPS administration in liver tissue indicating that its production is mainly responsible for liver injury. Zingerone treated liver cells showed significantly low levels of TNF- α suggesting less hepatotoxicity and tissue inflammation.

We also checked the mRNA expression levels for iNOS gene. Hyper expression of iNOS clearly indicated that oxidative damage to the liver is contributed by iNOS. iNOS expression is known to be enhanced by LPS leading to generation of nitric oxide radicals causing acute tissue injury [43]. Zingerone treatment significantly suppressed the mRNA levels of iNOS gene suggesting its antioxidant activity. Another inflammatory enzyme COX-2 is also activated by LPS stimulus. Previous reports have shown a potential role of tyrosine kinase in LPS promoter region that contain 24 transcriptional factor- binding sites, including those for nuclear factor-κB (NFκB) family, that appears to be essential in the enhanced COX-2 gene expression seen in macrophages exposed to endotoxin [44]. Cyclooxygenase-2 (COX-2) is an inducible enzyme of macrophages catalyzing the conversion of arachidonic acid to prostaglandins. Recent studies have suggested that increased levels of prostaglandins and cyclooxygenase activity and COX-2-derived bioactive lipids, including prostaglandin E2 (PGE2), are potent inflammatory mediators causing tissue injury. LPS induced very high mRNA expression of COX-2 (at 8 hour interval) and this probably may have led to increased production of prostaglandin E2 resulting in intense inflammation. Zingerone treatment significantly reduced mRNA expression of COX-2 which ultimately reduced the liver injury in treated animals. RelA, NF-κB2 are signaling molecules and regulate the expression of many inflammatory genes. Expression of these genes in the present study clearly indicated that these genes are involved in the signaling cascade and regulation of expression of inflammatory genes. Rel A and NF-κB2 gene expression was found to increase following LPS administration. Zingerone treatment significantly inhibited the expression level of these genes clearly indicating that zingerone was able to interfere with inter signaling pathways and suppress the hyper expression of important cell signaling molecules. Since, *P.aeruginosa* LPS showed maximum expression of all genes at 8 hour interval, this time period was chosen for observing the effect of zingerone on the expression of inflammatory markers. Expression of COX-2, TNF-α, iNOS, RelA, NF-κB2 and TLR4 was found to be highly suppressed by zingerone treatment at 8 h interval. Decrease in the mRNA expression levels in presence of zingerone indicated low amount of mRNA in the liver leading to decrease in protein levels with minimum LPS induced hepatotoxic effect. Zingerone has been found to be successful in reducing inflammation through multitargeted mechanism. In addition to free radical scavenging effects [21], reducing binding efficiently of LPS to LPS receptors and further interference with the activation of inflammatory signalling molecules. Results of the present study suggest that zingerone inhibited LPS-induced acute liver injury which was mediated via TLR4/NF-κB signaling pathway by suppressing the mRNA expression of inflammatory markers involved in this pathway. We hypothesize that zingerone may have altered the endotoxin receptor complex formation since ginger components particularly shogaols are known to inhibit TLR4 dimerization [45], [46]. Hence it may also have the potential to inhibit TLR4 dimerization or TLR4 and MD-2 complex formation. Both steps are necessary for the downstream signalling of the endotoxin induced expression of genes [45], [46]. The present study provides an insight on the impact of zingerone in suppressing inflammatory mediator production, reducing oxidative damage to liver tissue hence protecting liver from endotoxin induced injury. Understanding detailed mechanism of action of zingerone may lead to finding novel targets for suppression of LPS induced inflammation.

## Conclusions

Zingerone a nontoxic, inexpensive dietary natural compound with potent anti-inflammatory and pharmacological activities having no side effect showed hepatoprotective effect against endotoxin induced liver injury via scavenging free radicals and down regulating production of inflammatory mediators. This study opens different areas to venture zingerone as potential anti-inflammatory molecule for reducing endotoxin induced inflammation in *P. aeruginosa* infections as well as during antibiotic treatment.
